# Comparative Analysis of Radiological Evaluation and Early Functional Outcomes of Total Knee Arthroplasty Using an Accelerometer-Based Handheld Navigation System and Conventional Instrumentation: A Prospective Study

**DOI:** 10.7759/cureus.21039

**Published:** 2022-01-09

**Authors:** Nuthan Jagadeesh, Hiranya Kumar, Varma sarparaju, Vishwanath Shivalingappa

**Affiliations:** 1 Trauma and Orthopedics, Vydehi Institute of Medical Sciences and Research Centre, Bengaluru, IND

**Keywords:** accelerometer based navigation tka, knee society score, oxford knee score, hand held navigation system, radiological evaluation of knee arthroplasty, navigation system, knee arthroplasty

## Abstract

Background

An accelerometer-based handheld navigation system (HHNS) for total knee arthroplasty (TKA) does not require a large console needed for computer-assisted navigation systems and has been shown to decrease component malalignment in TKA. The study aimed to use HHNS with conventional instrumentation to compare the radiological evaluation and functional outcomes of TKA.

Materials and methods

This was a multi-surgeon, prospective, assessor-blinded comparative study of 122 patients undergoing unilateral TKA. We used a stratified randomized sampling method to select 35/48 patients undergoing TKA using a handheld navigation system and 35/74 patients undergoing TKA using conventional instrumentation and divided them into two groups: the HHNS group and (conventional) CONV group. Postoperative radiographic evaluation was based on the tibial and femoral alignment angle, posterior tibial slope, and tibiofemoral angle measured from full-length lower-limb anteroposterior and lateral views of the knee. The Oxford Knee Score (OKS) and Knee Society Score (KSS) with a two-year serial follow-up were used to evaluate functional outcomes.

Results

The mean tibial alignment angle and posterior tibial slope were 0.78° ± 1.27° and 4.38° ± 0.86°, respectively, in the HHNS cohort and 2.63° ± 1.54° and 2.12° ± 1.82°, respectively, in the CONV group (*p* < 0.001). There was no significant difference in the femoral alignment angles. The overall alignment using the mean tibiofemoral angle was 179.21° ± 1.82° in the HHNS group and 177.31° ± 2.18° in the CONV group (*p* = 0.002). There were no significant differences in the KSS and OKS at the two-year follow-up between the groups.

Conclusions

The use of HHNS in TKA significantly increased accuracy in limb and implant alignment, but there was no significant difference in the two-years functional outcomes.

## Introduction

Total knee arthroplasty (TKA) is an established procedure for treating advanced knee arthritis. Good surgical technique and instrumentation leading to accurate mechanical axis alignment and appropriate soft-tissue balancing are required not only for the long-term survival of implants but also for better functional outcomes [1-2].

Mechanical alignment guides developed to achieve accurate postoperative mechanical axis alignment would be systems based on intramedullary or extramedullary rods. Immediate postoperative component malposition influences overall limb alignment, the lifespan of the components, and overall function. Despite improved alignment guides, malalignment is still very much prevalent [3-6]. There have been significant degrees of errors in mechanical axis alignment (>3°) ranging from 22% to 35% for routine TKAs using conventional instrumentation [2]. Computer-assisted surgery (CAS) navigation, handheld navigation systems, and patient-matched instrumentation have been developed as alternatives to conventional instrumentation to minimize these errors.

An accelerometer-based handheld navigation system (HHNS) for TKA does not require a large console needed for CAS. This instrumentation is a palm-sized handheld navigation system for use in TKA. HHNS acts as a guide for the more accurate distal femur (varus/valgus) and proximal tibia cuts (posterior slope, varus/valgus). Thus, it is known to improve radiological limb alignment and avoid component malpositioning [7]. Although good radiological outcomes have been reported [8-13], the evidence on the clinical outcomes using this device is limited, and there have been few randomized studies. The primary objective of this study was to determine if HHNS, compared with conventional instrumentation, increases the accuracy of tibial and femur resection and, in turn, improves overall alignment. The secondary objective was to compare early functional outcomes evaluated by the self-reported Knee Society Score (KSS) and Oxford Knee Score (OKS) between the two groups.

## Materials and methods

Design

**Figure 1:**
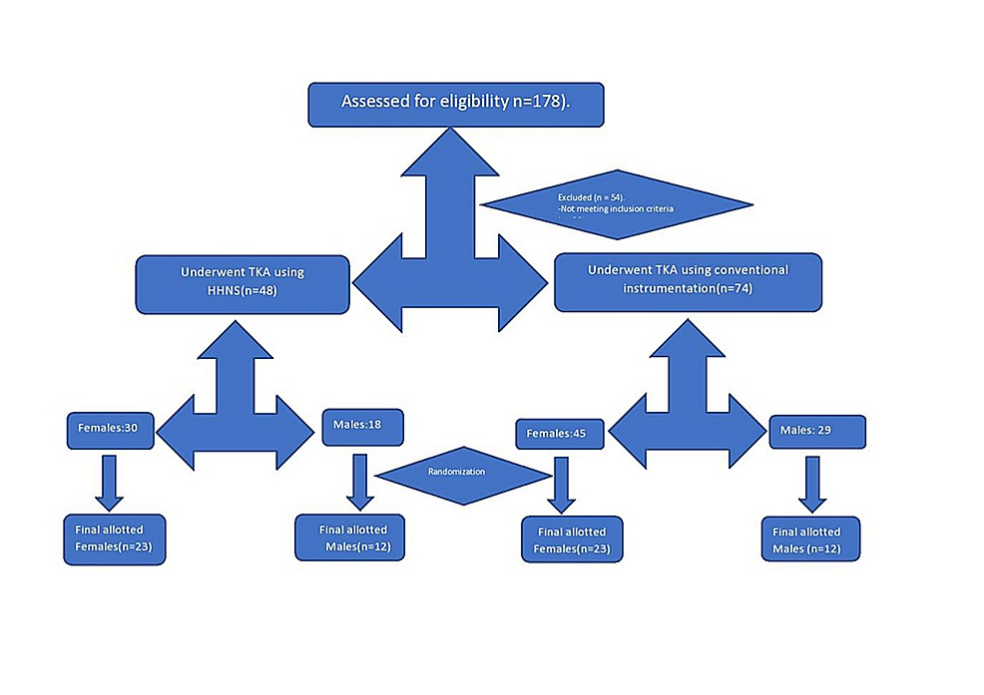
Methodology of patient enrolment

This multi-surgeon, assessor-blinded, prospective comparative study enrolled 178 patients with advanced primary osteoarthritis between July 2017 and December 2018 treated in our orthopedic department who were assessed for study eligibility after obtaining institutional ethics committee approval. Patients were excluded if they had secondary arthritis, such as rheumatoid arthritis, femoral or tibial bone defects, Fixed flexion deformity >10°, any source of active infection, or associated major hip or spine abnormalities. Revision surgeries, bilateral total knee replacement (TKR), surgeries requiring metal augments, or extended stems were also excluded. After excluding 54 patients, 122 patients undergoing TKR who provided informed consent and planned to undergo unilateral TKR were enrolled in the study. We used a stratified randomized sampling method and randomization computer software to select 35/48 patients planning to undergo TKR using a handheld navigation system (HHNS group) and 35/74 patients undergoing TKR using conventional instrumentation (CONV group). The patient selection methodology is shown in Figure 1. Full-length lower-limb (hip to ankle) anteroposterior and lateral views of knee roentgenograms in standing were used to measure preoperative lower-limb axis and varus/valgus deformity at the knee. A group of three senior orthopedic surgeons who specialized in replacement surgeries performed all the surgeries, and each of them had many years of experience with TKA using conventional and accelerometer-based instrumentation.

Surgical technique and radiological measurements

**Figure 2:**
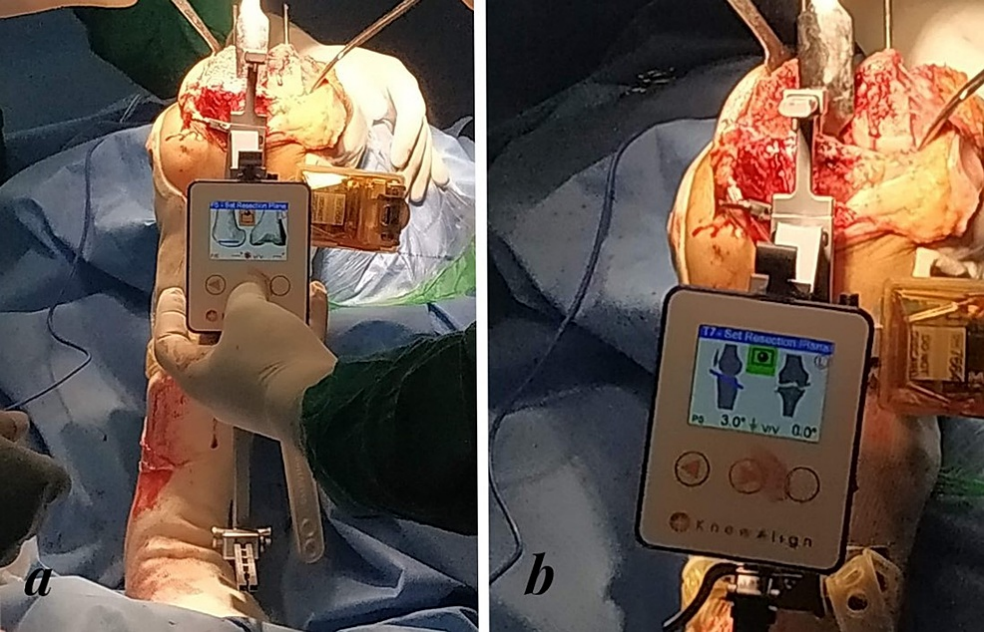
Use of KneeAlign 2 navigation system in a) femur resection b) tibial resection.

Patients allocated to the HHNS group underwent replacement surgery (midline incision, parapatellar arthrotomy) using the HHNS (KneeAlign 2) instrumentation to perform the proximal tibial resection and distal femur resection (Figure 2). The device comprises a display console and reference sensor, which use triaxial accelerometers to communicate with each other. The first step following exposure of the knee joint is to secure the micro block and sensor on the distal femur. The center pin is placed at the same site as the intramedullary guide rod in analog guides. The display console and reference sensor are attached to the femoral jig and cutting block, respectively. The next step is to calculate the femur center of rotation, which is done by maneuvering the leg in the adduction, and abduction of the hip and flexion and extension of the hip. The device predicts the appropriate cutting position concerning varus/valgus (coronal) and flexion/extension alignment of the component. The depth of resection is adjusted by moving the cutting block up/down concerning the distal femoral condyles. Once the final position of the cutting block is confirmed, it is locked in that position by two headless pins. The distal femur resection is then performed. The resection plane is calculated by the navigation unit, and the desired resection depth is set by the surgeon. The remaining steps of distal femur resection are the same as in a traditional TKA. The tibia is prepared next. There are two primary components of the tibial jig: the mobile component and a fixed component, which are assembled. The first step is to align the proximal guide with the medial one-third of the tibial tubercle and place the stylus over the anterior cruciate ligament footprint. The medial and lateral malleolus locations, as well as offset, are registered. The appropriate position cutting block of the tibial resection with appropriate varus/valgus alignment and the posterior tibial slope is determined and fixed with pins. The surgeon selects the acceptable resection depth before pinning the cutting block. The rest of the steps after tibial resection are similar to those of conventional TKR. Patients allocated to the CONV group underwent TKA using conventional instrumentation in which the extramedullary guide was used to perform the tibial resection and the intramedullary alignment guide was used for distal femur resection. Gap balancing was performed with the adequate soft-tissue release, and proper patellar tracking was ensured in all cases in both groups. The patients in both groups were implanted with a fixed-bearing, cemented, posterior cruciate ligament-substituting TKA.

**Figure 3:**
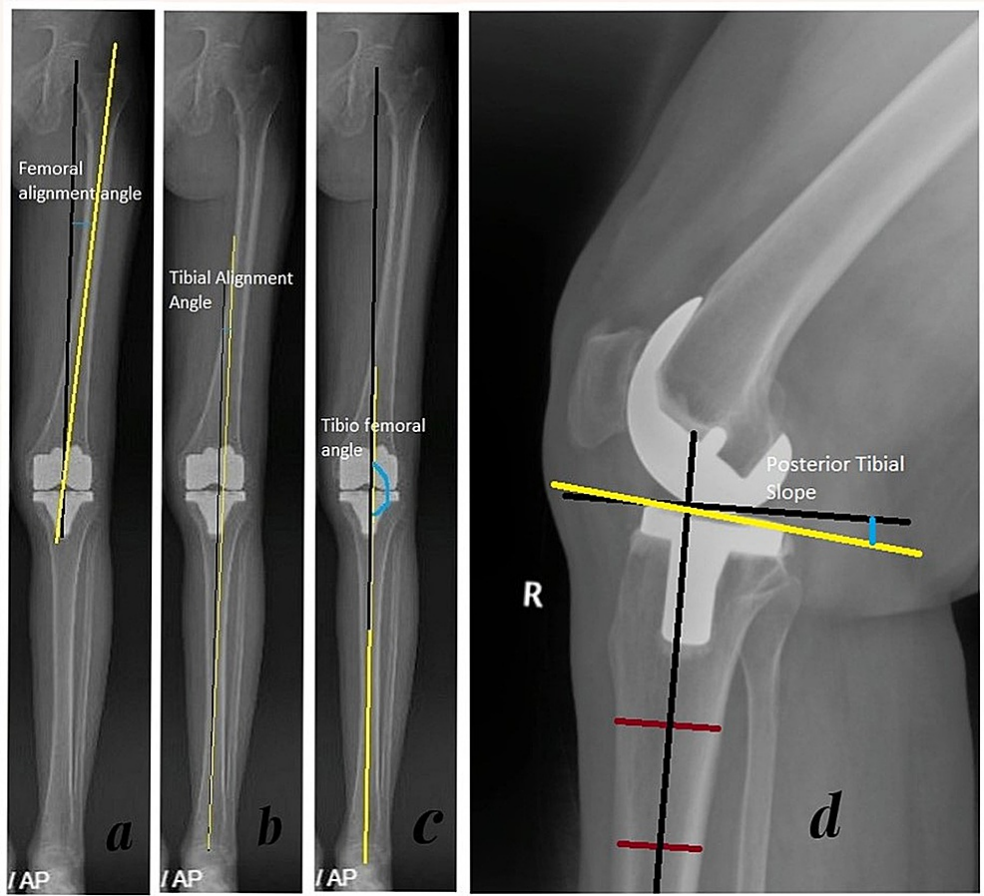
: Basis for calculation of parameters used for radiological evaluation. a) Femoral alignment angle: angle between the mechanical axis of the femur, i.e., the line joining the femoral-head center with the knee joint center, and the anatomical axis of the femur. b) Tibial alignment angle: angle between the mechanical axis and anatomical axis of the tibia. c) Tibiofemoral angle: angle between the mechanical axis of the femur and the mechanical axis of the tibia. c) Posterior tibial slope: angle between the line perpendicular to the mechanical axis of the tibia and the tangent along the upper aspect of the tibial component.

The target femoral coronal resection angle was 4º-6º distal femur valgus angle to an anatomical axis that corresponds to 0º mechanical varus/valgus alignment. The target tibial alignment angle was 0° ± 2° varus/valgus alignment. The target posterior tibial slope was 3° ± 2°. The target overall tibiofemoral angle was 0° ± 2° varus/valgus in all patients, which corresponds to 0º mechanical axis varus/valgus alignment. Full-length lower-limb (hip to ankle) anteroposterior and lateral views of the knee were taken at the first postoperative visit at two weeks for all patients. All radiographic measurements, i.e., the tibial alignment angle, femur alignment angle, posterior tibial slope, and tibiofemoral angle, were made by two orthopedic surgeons who were blinded to the technique used (Figure 3). By convention, all varus alignment measurements were assigned a positive value, and valgus alignment measurements were assigned a negative value. Slope measurements were negative for anterior slope and positive for posterior slope. Interobserver and intraobserver reliabilities between the radiological measurements were determined by the use of intraclass correlation coefficients (ICCs) of the implant alignment measurement for the femoral and tibial components. All patients on either group were treated by following the standard postoperative physiotherapy protocol, deep-vein thrombosis prophylaxis, and were discharged after two dressings on postoperative day five.

Sample size calculation

Sixty patients (30 per group) were required to have a 90% chance of detecting a decrease in the primary outcome variable from 13.5 in the control/conventional group to 11.2 in the experimental group, with an anticipated standard deviation (SD) of 2.71 (significance level of 5%).

The calculation was based on the formula:

n = f(α/2, β) × 2 × σ2 / (μ1 − μ2)2

where μ1 and μ2 are the mean outcomes in the HHNS and CONV groups, respectively, and σ is the SD.

Outcome measures

The OKS and KSS were assessed preoperatively at two weeks, six weeks, six months, one year, and two years after surgery for both patient groups and compared. The objective KSS was assessed by the orthopedic surgeons who were blinded to the procedure, whereas the functional KSS and OKS were self-reported by the patient. If the patient was unable to visit, especially because of the COVID-19 pandemic, the scores were assessed by telephonic consultation [13,14].

Statistical analysis

All data were analyzed in IBM Corp. Released 2013. IBM SPSS Statistics for Windows, Version 22.0. Armonk, NY: IBM Corp. and Microsoft Office 2007 (Microsoft Corp., Redmond, WA). All continuous variables were presented as the mean ± SD, and all categorical data were presented as the number and percentage. The unpaired t-test and Chi-square (χ2) test were used to analyze data between two continuous and categorical variables, respectively. The level of statistical significance was set to p < 0.05.

## Results

Comparison of demographic data, preoperative radiographic measurements, and clinical scores between the two groups did not show any significant differences. All patients had varus deformity preoperatively ranging from 7º to 18º. The mean preoperative tibiofemoral angle in the HHNS was 168.85º ± 8.21º versus 169.51º ± 6.74º in the CONV group (Table 1). The overall intra- and interobserver reliabilities determined by ICCs of the implant alignment measurement for the femoral and tibial components and tibiofemoral angles were good (ICC > 0.70). There were no intraoperative complications related to issues in the HHNS system.

**Table 1:** Preoperative demographics variables, such as age, sex, affected side, and preoperative tibiofemoral angle. * significant at p < 0.05

	HHNS Group	CONV Group	p value
Sex			
Male	12	12	NA
Female	23	23	NA
Side			
Right	16	14	0.485
Left	19	21	0.634
Age (years)	61.88	63.85	0.877
Preop tibiofemoral angle*	168.85	169.51	0.346

The mean tibial alignment angle was 0.78° ± 1.27° in the HHNS group versus 2.63° ± 1.54° (p < 0.001) in the conventional group, which was significant. Moreover, 91.4% of tibial components in the HHNS group were within the target value, whereas only 77.1% of tibial components in the conventional group were within the target range (Table 2).

**Table 2:** Comparison of the tibial alignment angle, femoral alignment angle, posterior tibial slope, tibiofemoral, KSS, and OKS between the HHNS and CONV groups. * significant at p < 0.05

Parameters	HHNS group (N = 35)		CONV group (N = 35)		Mean difference	95% CI		p value
	Mean	SD	Mean	SD		Lower	Upper	
Tibial alignment angle	0.78	1.27	2.63	1.54	1.85	0.28	3.04	<0.001*
Post-tibial slope	4.38	0.86	2.12	1.82	−2.26	−1.78	−0.36	0.005*
Femoral alignment angle	5.38	1.74	5.54	1.10	−0.16	−0.87	0.56	0.549
Tibiofemoral Angle	179.21	1.82	177.31	2.18	−1.90	−2.38	−0.38	<0.001*
KSS score - objective								
Preop	41.8	5.42	44.42	5.87	2.62	−4.62	5.87	0.803
6 weeks	67.78	4.37	66.64	4.21	1.14	−2.78	2.46	0.585
6 months	77.63	3.7	78.39	4.8	0.76	−1.78	1.85	0.321
1 year	92.43	2.41	91.21	2.34	1.22	−1.58	0.95	0.678
2 year	95.64	1.44	94.8	1.72	1.44	−0.75	0.82	0.812
KSS score - functional								
Preop	53.23	4.32	50.25	5.46	2.00	−0.16	2.98	0.826
6 weeks	66.37	3.46	64.83	3.26	1.54	−0.68	2.12	0.484
6 months	77.64	2.12	76.71	1.98	1.07	−1.26	0.36	0.243
1 year	91.15	1.6	91.30	2.0	−0.15	−0.28	0.94	0.781
2 year	94.22	1.60	94.21	1.61	0.01	−0.03	0.77	0.978
OKS score								
Preop	18.6	3.79	18.62	4.23	−0.02	−1.92	1.88	0.984
6 weeks	33.8	0.72	30.6	0.81	3.20	−0.57	0.17	<0.001*
6 months	38.9	1.24	37.7	0.90	0.19	−0.71	0.32	0.458
1 year	46.78	0.89	44.85	0.96	−0.07	−0.51	0.37	0.653
2 year	47.73	0.45	46.96	0.50	−0.03	−0.26	0.19	0.756

The mean femoral alignment angle was 5.38° ± 1.74° in the HHNS cohort versus 5.54° ± 1.10° (p = 0.64) in the conventional cohort (p = 0.64, not significant). However, 97.1% of the femoral components in the HHNS group were within the target value, whereas only 88.5% of the femoral components in the CONV group were within the target range (Table 2). There was a significant difference in the mean posterior tibial slope between the HHNS group (4.38° ± 0.86°) and CONV group (2.12° ± 1.82°) (p = 0.005) (Table 2). The overall alignment using the mean tibiofemoral angle was 179.21° ± 1.82° in the HHNS group and 177.31° ± 2.18° CONV group, which was significant (p = 0.002) (Table 2). The average follow-up period in our study was 22.3 ± 2.8 months (range: 15-32 months) in the HHNS group and 21.2 ± 3.9 months (range: 14-34 months) in the conventional group with a minimum follow-up period of ≥1 year (Table 2). At the two-year follow-up, both the objective and functional part of the KSS were similar between the HHNS group (95.6 ± 1.44 and 94.2 ± 1.6, respectively) and CONV group (94.8 ± 1.72 and 94.2 ± 1.61, respectively), but the differences were not significant. Although there was no difference in the OKS score at most intervals between the groups, there was a significant difference at six weeks (p < 0.001) (Table 2).

## Discussion

An HHNS uses accelerometers and gyroscopes to achieve alignment of the femur and tibial components. The system is similar to conventional TKA with an extramedullary jig for proximal tibial cuts, but there is no need for an intramedullary jig for distal femur cuts, which has a prominent role if a femoral diaphyseal deformity is present where an intramedullary jig cannot be used. Handheld navigational system TKA has provided accuracy in the placement of femoral and tibial components as well as the overall mechanical alignment of the limb [15,16]. The study aim was to evaluate the accuracy of the tibial and femur resection cut made using HHNS when compared with conventional instrumentation and whether it achieves better functional outcomes in the early follow-up period.

Numerous studies have concluded that implanting the components within ±3° of the mechanical axis of the lower limb not only helps implant survival but also provides better results functionally and improves the quality of life [17-20]. The postoperative tibiofemoral angle is the angle between the mechanical axis of the femur and tibia that would be a straight-line forming 180° with perfect neutral alignment. In their study of radiographic assessment of knee replacements using an accelerometer-based portable navigation device, H. Shoji et al. [21] showed that the mean tibiofemoral angle was 179.3° ± 2.6°, with just 10% outliers. In our study, the average tibiofemoral angle was 179.21° ± 1.82º in the HHNS group and 177.31° ± 2.18° in the CONV group, with a significant difference. The tibiofemoral angle outside ±3° of neutral alignment was only 6% in the HHNS group and 17% in the CONV group.

Berend et al. [17] evaluated the mechanism of tibial component failure and concluded that medial bone collapse caused by a >3° varus malalignment was the most common reason behind the tibial component revision. Therefore, methods of improving tibial component positioning in TKA are the most crucial factor that could prolong the survival of the implant [21]. A randomized controlled trial conducted by Denis Nam et al. [22] comparing the KneeAlign 2 accelerometer-based handheld navigational system with the conventional extramedullary guides reported a significant reduction of the number of “outliers” for tibial component alignment in both the coronal and sagittal planes. In their study, the mean tibial component coronal alignment was −0.6° ± 0.9° in the KneeAlign 2 cohort versus −0.9° ± 1.6° in the conventional cohort (p = 0.26) [22-23]. In our study, the mean tibial alignment angle was 0.78º ± 1.27º in the HHNS group and 2.63 ± 1.54º in the CONV group (p < 0.001). The percentage of tibial components placed within 2° of the intraoperative coronal alignment goal was 91.4% in the HHNS group and 77.1% in the CONV group.

Regarding sagittal alignment, there is extreme variability in the desired target of the posterior tibial slope. W.D. Bugbee et al. [24] chose the target to be either a 3° or 5° posterior slope, and the handheld system was 95.5% accurate in creating a posterior slope within ±3° of the intraoperative goal. Denis Nam et al. [22] reported that 95.0% of the KneeAlign 2 cohort and 72.1% of the conventional cohort were within ≤2° of 3º of the posterior slope in the sagittal plane. We targeted our posterior tibial slope to be between 3º and 6º, and the mean posterior tibial slope was 4.38º ± 0.86º in the HHNS group and 2.12º ± 1.82º in the CONV group (p = 0.005). A posterior tibial slope within the target range was achieved in 89.7% of the HHNS group and 74.1% of the CONV group.

Neutral alignment of the lower limb can be ensured by a perpendicular cut made to the tibial and femoral mechanical axis. Unless there is a deformity at the tibial shaft, the tibial cut made perpendicular to its anatomical axis corresponds to the mechanical axis of the tibia, but the same cannot be done on the femoral side as the anatomical axis does not correspond to the mechanical axis of the femur and makes a valgus angle of 4º-6º. R. Vaishya et al. [25] evaluated variation in the distal femoral valgus angle in an Indian population that had an average femoral valgus angle of 5.83º ± 0.64º and concluded that it is justifiable to use a fixed femoral valgus cutting angle in the patients undergoing TKA. Lotke et al. [26] evaluated the perfect position of knee implant in TKA and suggested that the femoral valgus angle should be 4º-6º, which corresponds to the neutral mechanical axis. We targeted the femoral alignment angle to be 4º-6º, and the mean femoral alignment angle was 5.38º ± 1.74º in the HHNS group and 5.54º ± 1.10º in the CONV group, but the difference was not significant.

Good lower-limb alignment avoids an abnormal load distribution, which would help in overall good clinical outcome. Although numerous studies have established the role of HHNS in better alignment of components, its effect on functional outcome is limited. Lorio et al. [10] concluded that the use of KneeAlign 2 resulted in accurate tibial alignment and better self-reported clinical outcomes postoperatively. In our study, both the objective and functional KSS were similar in both the HHNS group (95.6 and 94.2, respectively) and CONV group (94.8 and 94.2, respectively), and the differences were not significant. Although the self-reported OKS was significantly better at six weeks postoperatively in the HHNS group, the scores in the CONV group improved with time. At two years of follow-up, the OKS values were similar between the HHNS group and the CONV group, with no significant difference. We concluded that patients who underwent HHNS tolerated the surgical insult better than those in the the CONV group because the OKS at six weeks was significantly better. However, the postoperative clinical outcomes in TKA using the HHNS were similar to those for conventional TKR assessed by all three scoring systems at the two-year follow-up.

A limitation of this study was that it was a multi-surgeon study with a short follow-up period. The influence of several radiological parameters, such as condylar offset and rotational alignment of the implant, on the long-term survival of the implant, were not discussed. Moreover, we randomly selected a subgroup of patients among all of the included cases to reduce selection bias, whereas it would have been ideal to allocate the patients before the operation to respective groups. To summarize, although there was no significant variation in the accuracy of the distal femur cuts, this study demonstrated that HHNS significantly reduced the malalignment in proximal tibial cuts in TKA. Thus, the overall mechanical axis of the lower limb was restored better with HHNS than with conventional instrumentation.

## Conclusions

The use of HHNS in TKA significantly increased accuracy in limb and implant alignment, with no added advantage of improved functional outcomes at the two-year follow-up when compared with those of conventional instrumentation. When compared with conventional methods, HHNS had several advantages: the instrumentation is simpler, it is single-use and patient-specific, performs calculations, and recommends bony cuts based on an individual patient’s anatomy. No preoperative preparation time is needed, nor are there any intraoperative line of sight issues. In addition, HHNS does not require intramedullary violation of the femur canal and could be used for patients with a deformity of the femur. The device combines the benefit of avoiding a large console, such as those in computer-assisted navigational or robotic TKA, and avoids intramedullary violation as in conventional TKA. Moreover, there is no need for huge initial costs as required in robotic TKA or computer-assisted navigational TKA, which makes HHNS an attractive and feasible alternative.

## References

[1] Anderson KC, Buehler KC, Markel DC (2005). Computer assisted navigation in total knee arthroplasty: comparison with conventional methods. J Arthroplasty.

[2] Blakeney WG, Khan RJ, Wall SJ (2011). Computer-assisted techniques versus conventional guides for component alignment in total knee arthroplasty: a randomized controlled trial. J Bone Joint Surg Am.

[3] Koh YG, Hong HT, Lee HY, Kim HJ, Kang KT (2021). Influence of variation in sagittal placement of the femoral component after cruciate-retaining total knee arthroplasty. J Knee Surg.

[4] Arbab D, Reimann P, Brucker M, Bouillon B, Lüring C (2018). Alignment in total knee arthroplasty - a comparison of patient-specific implants with the conventional technique. Knee.

[5] Laskin RS (1984). Alignment of total knee components. Orthopedics.

[6] Ritter MA, Faris PM, Keating EM, Meding JB (1994). Postoperative alignment of total knee replacement. Its effect on survival. Clin Orthop Relat Res.

[7] Huang EH, Copp SN, Bugbee WD (2015). Accuracy of a handheld accelerometer-based navigation system for femoral and tibial resection in total knee arthroplasty. J Arthroplasty.

[8] Reed MR, Bliss W, Sher JL, Emmerson KP, Jones SM, Partington PF (2002). Extramedullary or intramedullary tibial alignment guides: a randomised, prospective trial of radiological alignment. J Bone Joint Surg.

[9] Nam D, Dy CJ, Cross MB, Kang MN, Mayman DJ (2012). Cadaveric results of an accelerometer based, extramedullary navigation system for the tibial resection in total knee arthroplasty. Knee.

[10] Iorio R, Mazza D, Drogo P (2015). Clinical and radiographic outcomes of an accelerometer-based system for the tibial resection in total knee arthroplasty. Int Orthop.

[11] Ueyama H, Minoda Y, Sugama R (2019). An accelerometer-based portable navigation system improved prosthetic alignment after total knee arthroplasty in 3D measurements. Knee Surg Sports Traumatol Arthrosc.

[12] Nam D, Weeks KD, Reinhardt KR, Nawabi DH, Cross MB, Mayman DJ (2013). Accelerometer-based, portable navigation vs imageless, large-console computer-assisted navigation in total knee arthroplasty: a comparison of radiographic results. J Arthroplasty.

[13] Roos EM, Roos HP, Lohmander LS, Ekdahl C, Beynnon BD (1998). Knee injury and osteoarthritis outcome score (KOOS)-development of a self-administered outcome measure. J Orthop Sports Phys Ther.

[14] Dawson J, Fitzpatrick R, Murray D, Carr A (1998). Questionnaire on the perceptions of patients about total knee replacement. J Bone Joint Surg.

[15] Nam D, Cross M, Deshmane P, Jerabek S, Kang M, Mayman DJ (2011). Radiographic results of an accelerometer-based, handheld surgical navigation system for the tibial resection in total knee arthroplasty. Orthopedics.

[16] Nam D, Nawabi DH, Cross MB, Heyse TJ, Mayman DJ (2012). Accelerometer-based computer navigation for performing the distal femoral resection in total knee arthroplasty. J Arthroplasty.

[17] Berend ME, Ritter MA, Meding JB (2004). Tibial component failure mechanisms in total knee arthroplasty. Clin Orthop Relat Res.

[18] Ritter MA, Davis KE, Meding JB, Pierson JL, Berend ME, Malinzak RA (2011). The effect of alignment and BMI on failure of total knee replacement. J Bone Joint Surg.

[19] Attar FG, Khaw FM, Kirk LM, Gregg PJ (2008). Survivorship analysis at 15 years of cemented press-fit condylar total knee arthroplasty. J Arthroplasty.

[20] Ensini A, Catani F, Leardini A, Romagnoli M, Giannini S (2007). Alignments and clinical results in conventional and navigated total knee arthroplasty. Clin Orthop Relat Res.

[21] Shoji H, Teramoto A, Suzuki T, Okada Y, Watanabe K, Yamashita T (2018). Radiographic assessment and clinical outcomes after total knee arthroplasty using an accelerometer-based portable navigation device. Arthroplast Tod.

[22] Nam D, Cody EA, Nguyen JT, Figgie MP, Mayman DJ (2014). Extramedullary guides versus portable, accelerometer-based navigation for tibial alignment in total knee arthroplasty: a randomized, controlled trial: winner of the 2013 HAP PAUL award. J Arthroplasty.

[23] Matsumoto K, Ogawa H, Fukuta M, Mori N, Akiyama H (2018). Comparative study for alignment of extramedullary guides versus portable, accelerometer-based navigation in total knee arthroplasty. J Knee Surg.

[24] Bugbee WD, Kermanshahi AY, Munro MM, McCauley JC, Copp SN (2014). Accuracy of a hand-held surgical navigation system for tibial resection in total knee arthroplasty. Knee.

[25] Vaishya R, Vijay V, Edomwonyi EO, Agarwal AK (2018). Fixed distal femoral valgus cutting angle is still justifiable in total knee replacement. J Clin Orthop Trauma.

[26] Lotke PA, Ecker ML (1977). Influence of positioning of prosthesis in total knee replacement. J Bone Joint Surg Am.

